# Guidance for Grading the Evidence in Quantitative Umbrella Reviews

**DOI:** 10.1111/jebm.70137

**Published:** 2026-06-25

**Authors:** Hanan Khalil, Ritin Fernandez, Patraporn Bhatarasakoon, Kim Sears, Dawid Piper, Jennifer Stone, Cindy Stern, Kate Kynoch

**Affiliations:** ^1^ Department of Public Health School of Psychology and Public Health La Trobe University Melbourne Australia; ^2^ School of Nursing and Midwifery University of Newcastle Newcastle Australia; ^3^ Faculty of Nursing Chiang Mai University Chiang Mai Thailand; ^4^ School of Nursing Queen's University Kingston Canada; ^5^ Faculty of Health Sciences Brandenburg Brandenburg Medical School (Theodor Fontane) Neuruppin Germany; ^6^ JBI Faculty of Health and Medical Sciences The University of Adelaide Adelaide Australia; ^7^ Health Evidence Synthesis Recommendations and Impact (HESRI) School of Public Health The University of Adelaide Adelaide Australia; ^8^ Nursing Research Center and the Queensland Center for Evidence‐Based Nursing and Midwifery Mater Health Services Brisbane Australia

**Keywords:** credibility assessment, GRADE, umbrella reviews

## Abstract

**Background:**

Umbrella reviews (URs) synthesize evidence across multiple systematic reviews and meta‐analyses to inform decision‐making. However, evaluating and integrating evidence certainty across overlapping and sometimes conflicting meta‐analyses remains a major methodological challenge of URs, limiting the reliability of conclusions.

**Objective:**

To compare evidence evaluation frameworks for URs, assess their suitability for different study types, and provide guidance for managing overlapping evidence, conflicting certainty ratings, and forming overall conclusions.

**Methods:**

We mapped published UR methods to identify current practices and gaps. Frameworks were compared across three dimensions: applicability to study types (interventional, causal observational, and descriptive observational), scalability and reproducibility, and capacity to handle methodological challenges specific to URs. Recommendations were developed through expert consensus and validated via case studies across diverse domains.

**Results:**

Grading of Recommendations Assessment, Development and Evaluation (GRADE) is widely accepted for interventional evidence but shows limited applicability for observational URs due to subjective judgments and reproducibility issues. Credibility frameworks, based on predefined statistical thresholds, offer greater scalability and reproducibility but require adaptation for different study types. Key challenges include: (1) managing overlapping primary studies, (2) resolving conflicting certainty ratings between high‐quality reviews, and (3) ensuring transparent evidence integration.

**Conclusions:**

Framework selection should be tailored to study type and context, with credibility frameworks better suited for observational evidence and GRADE optimal for interventional studies. Systematic approaches to overlapping evidence and conflicting ratings are essential for UR validity. We provide practical recommendations for framework selection, strategies for common challenges, and enhanced reporting standards to improve transparency and reproducibility.

## 1 Introduction

In recent years, the number of published systematic reviews has surged, increasing the demand for umbrella reviews (URs), which synthesize findings from multiple systematic reviews on a broad topic [1]. This demand is driven by several factors: (i) fragmentation of evidence across thousands of reviews, (ii) concerns about repeatability and overlap of systematic reviews, (iii) increasing complexity of decision‐making requiring broad evidence bases, and (iv) growing recognition of URs within the evidence ecosystem, alongside field synopses and overviews of reviews [2]. Despite their critical role, there is currently no universally accepted standard for assessing certainty of evidence in URs. This gap has prompted calls for transparent, validated frameworks tailored specifically to the UR context.

A scoping review by Sadoyu et al. systematically examined 99 non‐interventions URs published between 2010 and 2021 to explore how certainty of evidence is assessed [3]. The review found that only 56.6% of URs evaluated evidence certainty, with the majority (80.4%) applying the “criteria for credibility assessment.” In contrast, only 14.3% used the Grading of Recommendations Assessment, Development and Evaluation (GRADE) approach. Interestingly, URs published in high‐impact journals were significantly more likely to assess certainty, suggesting a link between methodological rigor and publication standards. The study also noted that the evaluation of certainty of evidence remained underdeveloped and inconsistently applied across URs. Moreover, we undertook a study mapping 100 UR methodologies and found 52% of URs did not assess the certainty of the evidence [1]. Our sample included URs informing health and a mixture of experimental, observational, and mixed studies. Of those that did assess certainty, most used the GRADE approach (*n* = 39, 39%). Other approaches included Confidence in the Evidence from Reviews of Qualitative research (CerQual) (*n* = 4, 4%) and NutriGrade for nutrition‐related meta‐analyses that uses nine domains for a score out of 10 points and more flexible than GRADE (*n* = 2, 2%). Eight URs (8%) referred to using other individual methods.

Given the increasing influence of URs on evidence‐informed decision‐making and policy‐driven evidence, the lack of methodological guidance is a notable gap. The literature supports the need for a uniform approach to assessing and reporting evidence certainty, tailored to the type of studies synthesized. The aim of this study is to systematically compare evidence evaluation frameworks for URs, clarify their suitability for different study types, and provide methodological guidance for handling evidence certainty and reaching overall conclusions at the UR level.

## 2 Methods

This study employed a multi‐faceted methodological approach comprising four components: a structured mapping of published UR methods, a systematic comparison of evidence frameworks across three key dimensions, purposive analysis of five representative case studies, and consultation with eight expert methodologists to develop evidence‐based recommendations for UR evaluation. All authors are members of the JBI UR methodology group and refined the content iteratively across three consensus meetings.

The present work constitutes a targeted, expert informed methodological synthesis, drawing on a previously published structured mapping as its evidentiary base [1]. It does not constitute a new scoping or systematic review; rather, it applies systematic comparative and analytical methods to already‐mapped data, supplemented by case study analysis and expert consultation. The initial mapping, published by our team, systematically identified and characterized data analysis and presentation methods in URs, encompassing search strategies, eligibility criteria, screening and selection processes, and data extraction protocols [1]. The present study applied the same rigorous approach, focused specifically on frameworks for grading certainty of evidence in quantitative URs.

The identified frameworks were then compared across three dimensions determined a priori: (1) applicability to study types—interventional, causal observational, and descriptive observational; (2) scalability and reproducibility; and (3) capacity to handle methodological challenges specific to URs. These dimensions were selected on the basis of the mapping findings, the existing literature on evidence grading in observational research, and preliminary expert input, and were agreed upon by the full author team. They were prioritized because they capture the practical barriers most consistently reported in the literature on evidence evaluation in URs. Other potentially relevant dimensions—including capacity to accommodate heterogeneity in eligibility criteria across included reviews—were considered but determined to fall within the scope of the three primary dimensions.

Five case studies were selected to validate and illustrate the recommendations, chosen to represent diversity across clinical domains, study designs, and framework applications. Selection criteria included relevance to UR methodology, representation of both interventional and observational evidence, and availability of sufficient grading detail. Each case study was analyzed using a standardized coding framework addressing framework application, handling of heterogeneity and bias, and the traceability of analytic findings to final recommendations [2]. Consensus recommendations were formed after analysis of the results.

## 3 Results

### 3.1 Methods for Assessing the Certainty of Evidence for URs of Quantitative Studies

Before describing individual frameworks, a brief conceptual clarification is warranted, as several related but distinct constructs appear throughout this literature. Statistical credibility, as operationalized in credibility classification frameworks, refers to the strength and robustness of an observed association based on quantitative thresholds applied to meta‐analytic outputs (e.g., *p* value, sample size, heterogeneity, and prediction intervals) [4, 5]. It is a measure of statistical confidence in the reported association and does not directly incorporate judgements about study design, risk of bias at the primary study level, indirectness, or clinical relevance. Certainty of evidence, as conceptualized in the GRADE framework, refers to confidence in the effect estimate—that is, the degree to which the true effect is likely to lie close to the estimated effect and is informed by multiple methodological domains, including risk of bias, inconsistency, indirectness, imprecision, and publication bias [6, 7, 8]. Clinical or policy decision certainty represents a further distinct construct, reflecting the confidence with which evidence can be used to guide a recommendation or action, incorporating not only the certainty of evidence itself but also its applicability, the magnitude of benefit and harm, values and feasibility. These constructs are not equivalent and should not be used interchangeably. Throughout this paper, the terms “credibility” and “statistical credibility assessment” refer specifically to the first construct (quantitative classification of meta‐analytic associations), while certainty of evidence is used in the GRADE sense unless otherwise specified.

Several methodological frameworks have been developed to assess the certainty of evidence in URs, particularly those synthesising observational studies [3, 9]. These approaches differ in their assumptions, data requirements, and intended applications. This section outlines the main methods currently used: the *GRADE approach*, the *credibility assessment framework*, and *other tailored classification systems*. Each is described in detail, highlighting its suitability for different types of evidence and review objectives.

#### 3.1.1 The GRADE Approach

GRADE was developed for intervention evidence and remains widely adopted by health organizations (World Health Organization, National Institute for Health and Care Excellence, National Health and Medical Research Council, and Cochrane). Its structured domains such as risk of bias, inconsistency, indirectness, imprecision, and publication bias—support nuanced interpretation when detailed study‐level data are accessible [7, 8].

Challenges for URs include incomplete reporting of primary study details within included meta‐analyses and the resource intensity of applying GRADE across large bodies of observational associations. Nonetheless, where interventional content is present or guideline translation is the goal, GRADE provides decision‐relevant outputs [1].

#### 3.1.2 Statistical Credibility Assessment

The credibility assessment framework is a structured, reproducible method commonly used in URs to evaluate the strength of evidence derived from meta‐analyses of observational studies. It is particularly useful when GRADE is less applicable due to the absence of individual study‐level data or the predominantly non‐randomized nature of the evidence. This approach enables reviewers to classify URs of observational studies into levels such as convincing, highly suggestive, suggestive, weak, or not significant, based on pre‐defined statistical criteria [3, 10]. Credibility frameworks classify exposure–outcome associations using predefined statistical thresholds (e.g., *p* value cut‐offs, sample size, heterogeneity, prediction intervals, small‐study effects, excess significance). They are reproducible and scalable for large observational corpora, particularly when study‐level details are limited.

Synthesizing credibility or certainty ratings across multiple meta‐analyses is a key challenge in URs. There are a few considerations that need to be taken into account. For example, overlap in primary studies across meta‐analyses must be quantified (e.g., using the corrected covered area statistic) to avoid double‐counting. Table 1 presents direct comparison between GRADE and credibility framework.

**TABLE 1:** Comparison of GRADE and credibility assessments.

Aspect	GRADE	Credibility framework
Primary purpose	Inform clinical/policy recommendations with methodological appraisal	Classify statistical robustness across many observational associations
Core inputs	Study‐level details and domain judgements (risk of bias, indirectness, etc.)	Meta‐analytic outputs: effect size, CI, *p*, *I* ^2^, prediction interval, and bias tests
Strengths	Nuanced, context‐aware; widely accepted for interventions; decision‐oriented	Reproducible, scalable; aligns with reported statistics; efficient for large datasets
Limitations	Resource‐intensive; may underweight strong observational signals when details are sparse	Risk of over‐reliance on statistical significance; limited capture of systematic bias
Best suited for	Interventional URs; guideline development and clinical pathways	Predominantly observational URs; epidemiology and exposure–outcome mapping
Handling heterogeneity	Judgement‐based inconsistency domain with narrative and quantitative synthesis	Quantitative (*I* ^2^, prediction intervals); requires nuanced interpretation beyond thresholds
Treatment of non‐significant results	Consider imprecision, power, and context within certainty ratings	Classify separately; emphasize uncertainty rather than binary dismissal

Abbreviations: CI, confidence interval; GRADE, Grading of Recommendations Assessment, Development and Evaluation; URs, umbrella reviews.

#### 3.1.3 Other Methods

Other methods have been used by researchers to assess certainty of the evidence [9, 11]. In the UR by Avşar et al., the authors assessed the certainty of evidence regarding the health impacts of smoking during pregnancy (SDP) and the postpartum period using a causal link analysis rather than applying the GRADE framework [11]. This decision reflected the nature of the data, which consisted primarily of meta‐analyses of observational studies with a wide range of outcomes and populations. The authors developed a structured classification based on the number of included studies and consistency of findings across reviews to evaluate the strength of the associations between SDP and various maternal and infant health conditions. Evidence was classified into four groups based on strength and consistency. Strong evidence included Group 1 (conditions supported by a single systematic review with ≥8 studies) and Group 2 (conditions supported by multiple systematic reviews with consistent findings). Weak evidence included Group 3 (conditions with conflicting findings across multiple systematic reviews) and Group 4 (conditions supported by only one systematic review with <8 studies) [11].

This classification was modified from 5 out of 9 established criteria from Hill's causality framework: strength, consistency, specificity, temporality, and plausibility [12]. Dose–response associations were also considered as supportive evidence. Although this approach does not explicitly quantify the overall certainty of evidence in the same way as GRADE, it allowed the authors to transparently group conditions by the robustness and consistency of the evidence base, particularly given the volume and heterogeneity of the included literature. The approach was well suited to the broad scope of the UR and provided a practical method for prioritizing conditions with the strongest support for a causal link to SDP. This approach represents a hybrid credibility framework combining systematic evidence synthesis with formal causal inference criteria, providing objective assessment and contextual clinical interpretation.

Another methodology used in the UR by Blume et al. assessed the credibility of evidence linking nurse staffing levels to nursing‐sensitive patient outcomes (NSPOs) using a structured, custom evidence grading system tailored for URs of observational literature [13]. Rather than applying GRADE, they developed a three‐tiered framework—high, moderate, and low—based on the proportion of significant findings across systematic reviews, weighted by the number of primary studies.

To classify the strength of evidence for each NSPO, the authors first coded associations as “significant” (Y = 1) if at least two‐thirds of the cited primary studies reported a statistically significant relationship, if a meta‐analysis showed a significant result, or if the review concluded there was a moderate or stronger association. Each literature review was then weighted according to how many primary studies it included per outcome (weight of 3 for >5 studies, 2 for 3–5 studies, and 1 for 1–2 studies). NSPOs were classified as high strength if ≥2/3 of the weighted systematic reviews found significant associations and included at least three primary studies; moderate strength if ≥1/3 but <2/3 of the reviews found significance; and low strength otherwise. Sensitivity analyses using alternative thresholds confirmed the robustness of this approach. This grading method allowed the authors to transparently summarize the cumulative evidence while accounting for both study quantity and consistency across reviews. It was well‐suited to the heterogeneous, correlational data included in their UR [13]. This represents a simplified strength‐of‐evidence framework focusing on consistency and significance of associations across multiple systematic reviews, rather than the more complex multi‐criteria approaches used in frameworks like GRADE.

### 3.2 Case Studies

The following case studies show practical application of credibility assessment frameworks and GRADE in URs across a variety of clinical and public health contexts. Together, these examples demonstrate the practical application of grading methods and highlight the rationale for selecting context‐specific approaches to evidence evaluation, as shown in Table 2.

**TABLE 2:** Summary of case studies: credibility and certainty assessment methods.

Case study	Type of studies included	Credibility assessment used	GRADE used
Physical activity and psychosocial outcomes (Purgato et al., 2024) [14]	Observational and interventional (RCTs)	Statistical credibility grading (Classes I–IV)	Yes—applied to RCTs
Periodontitis and NCDs (Rodríguez‐Medina et al., 2025) [15]	Observational (cohort, case‐control, and cross‐sectional)	Effect size classification (none, weak, moderate, and strong)	Yes—applied or retrospectively assessed
Air pollution and mental health (Radua et al., 2024) [16]	Observational (longitudinal and cross‐sectional)	Statistical credibility grading (Classes I–IV) using metaumbrella R package	No
Psychotropic safety in pregnancy (Fabiano et al., 2025) [17]	Observational (cohort and case‐control)	Statistical credibility grading (Classes I–IV) using metaumbrella R package	No
Comorbidities in bipolar disorder (Kang et al., 2024) [18]	Observational (cohort, case‐control, and cross‐sectional)	Statistical credibility grading (Classes I–IV) with re‐analyses	No

Classes I–IV refer to classification levels based on a statistical credibility grading framework. Class I (Convincing): the highest credibility level, requires *p* < 10^−^^6^, more than 1000 cases, significance in the largest study, *I* ^2^ below 50%, a prediction interval that excludes the null, and no evidence of bias. Class II (Highly suggestive): strong statistical evidence (*p* < 10^−^^6^, more than 1000 cases) but not all consistency or bias criteria are met. Class III (Suggestive): *p* < 0.001 with sufficient sample size but not meeting the higher thresholds of Class I or II. Class IV (Weak): statistically significant (*p* < 0.05) but not meeting the criteria for Class I, II, or III. High/Moderate/Low/Very Low refer to the four GRADE certainty levels.

Abbreviations: GRADE, Grading of Recommendations Assessment, Development and Evaluation; NCDs, non‐communicable diseases; RCTs, randomized controlled trials.

#### 3.2.1 Physical Activity as an Effective Therapeutic Strategy for Improving Psychosocial Outcomes in Children and Adolescents

Purgato et al. applied two complementary frameworks to assess the strength and certainty of evidence regarding physical activity interventions for psychosocial outcomes in children and adolescents: a quantitative credibility assessment and the GRADE approach [14].

In the reviewed article, the authors applied both the GRADE approach and a credibility classification framework to assess the certainty of evidence derived from meta‐analyses. These two methodologies yielded remarkably different conclusions.

The review found that while some associations were judged as “highly credible” using the statistical credibility criteria, they were simultaneously rated as “low” or “very low” certainty by GRADE. This divergence highlights a key limitation: credibility frameworks may overestimate the strength of evidence by focusing on statistical robustness alone, whereas GRADE integrates a broader perspective that incorporates methodological quality and applicability to real‐world decision‐making. These findings suggest that although both tools offer valuable insights, they serve different purposes—GRADE being more aligned with clinical guideline development, and credibility assessments better suited for summarizing statistical consistency in URs.

#### 3.2.2 Bidirectional Association Between Periodontitis and Noncommunicable Diseases

Rodríguez‐Medina et al. evaluated the bidirectional association between periodontitis and noncommunicable diseases (NCDs) using two complementary approaches to assess the strength and certainty of the evidence. The review synthesized findings from 24 systematic reviews and meta‐analyses, which primarily included observational studies such as cohort, case‐control, and cross‐sectional designs. To assess the magnitude of associations, the authors applied effect size criteria based on the classification system by Rosenberg and Handler, categorizing the strength of associations as none (odds ratio [OR], risk ratio [RR], or hazard ratio [HR] between 1.0 and 1.2), weak (1.2–1.5), moderate (1.5–3.0), or strong (3.0–10.0). This system was used to evaluate the strength of observed links between periodontitis and 32 NCDs, including cardiovascular disease, preterm birth, and cognitive impairment [15].

To assess the certainty of the evidence, the authors applied the GRADE approach. Where systematic reviews had already included GRADE assessments, those were retained; otherwise, the authors retrospectively applied GRADE using data reported in the original reviews. Given the predominance of observational evidence and issues such as heterogeneity and wide confidence intervals, most associations were graded as having low to very low certainty. The rationale for combining both effect‐size classification and GRADE was to offer a transparent, dual‐layered evaluation—capturing both the statistical strength of associations and the methodological reliability of the evidence linking oral and systemic health [15].

#### 3.2.3 Impact of Air Pollution and Climate Change on Mental Health Outcomes

In the UR by Radua et al., the authors synthesized findings from 32 systematic reviews with meta‐analyses, encompassing 284 primary observational studies (231 longitudinal and 53 cross‐sectional), to examine the association between air pollution and climate change hazards and mental health outcomes [16]. The review applied a credibility assessment framework to evaluate the strength of evidence for 237 exposure‐outcome associations. These associations covered a range of mental health outcomes—including mental disorders, suicidal behaviour, and mental health care access—and exposures such as PM_2.5_, carbon monoxide, nitrogen oxides, temperature, and cyclone events [16].

The strength of the evidence for each association was graded into four levels: convincing (Class I), highly suggestive (Class II), suggestive (Class III), and weak (Class IV), following established statistical criteria adapted from prior UR literature [5, 16]. These methods were operationalized using the metaumbrella R package, developed by the authors' team to automate credibility classification [4]. Unlike some other URs, the authors did not apply GRADE, instead choosing a fully statistical, bias‐sensitive framework suited to synthesizing observational environmental health data. Sensitivity analyses restricted to longitudinal studies were also conducted to confirm temporal relationships, further reinforcing the credibility of Classes I and II findings. This robust dual approach allowed the authors to identify actionable and globally relevant evidence linking environmental exposures to adverse mental health outcomes.

#### 3.2.4 Safety of Psychotropic Medications in Pregnancy

Fabiano et al. examined the association between psychotropic medication use during pregnancy and adverse health outcomes in pregnant individuals and their offspring. The authors synthesized data from 21 meta‐analyses, which encompassed 242 primary observational studies—including 206 cohort and 36 case‐control studies—covering over 17 million participants. The review focused on a range of psychotropic drug classes, including antidepressants, antipsychotics, mood stabilizers, benzodiazepines, and opioid maintenance therapies, and their associations with outcomes such as preterm birth, small for gestational age, congenital malformations, and neurodevelopmental effects [17].

To assess the strength of evidence, the authors applied a quantitative credibility assessment framework. Importantly, GRADE was not used in this review. Instead, the authors used the metaumbrella R package to standardize effect sizes (e.g., converting ORs, RRs, HRs, and standardized mean differences [SMDs] to equivalent ORs) and conduct the credibility assessments [4].

The review found that no associations met criteria for convincing or highly suggestive evidence. Only five (8%) reached the threshold for suggestive evidence, including associations between antidepressant or paroxetine use and outcomes such as preterm birth and congenital malformations. An additional 16 associations (24%) were supported by weak evidence, while the majority (68%) were statistically non‐significant. The rationale for this credibility‐based approach was grounded in the lack of high‐quality RCT data—owing to ethical constraints in pregnancy research—and the need for a reproducible, bias‐sensitive framework suitable for evaluating evidence from large‐scale observational data. This makes it one of the first URs to quantify the safety of psychotropics in pregnancy using a standardized grading system that accounts for confounding by indication and statistical rigor.

#### 3.2.5 Comorbid Physical Health Outcomes in Patients With Bipolar Disorder

In the UR by Kang et al., the authors investigated the association between bipolar disorder (BD) and various comorbid physical health outcomes by synthesizing data from 12 meta‐analyses comprising 145 original observational studies and over 60 million participants across 29 countries. The included studies spanned multiple observational designs, including cohort, case‐control, and cross‐sectional studies. Fourteen unique health outcomes were examined, including neurological, cardiovascular, metabolic, oncological, respiratory, gastrointestinal, infectious, and pain‐related conditions. The authors excluded psychiatric comorbidities to focus specifically on physical health outcomes [18].

To evaluate the certainty of evidence, Kang et al. applied a credibility classification framework, rather than the GRADE system as mentioned above by Fabiona et al. This grading considered several criteria: number of participants (>20,000), statistical significance (e.g., *p* < 10^−^^6^ for Class I/II), heterogeneity (*I* ^2^ < 50%), 95% prediction intervals excluding the null, and absence of small‐study effects or excess significance bias. The authors also applied multiple re‐analysis techniques (e.g., DerSimonian‐Laird and Hartung‐Knapp‐Sidik‐Jonkman models) and validated findings using tools such as Egger's test, *p*‐curves, and evidence mapping [19, 20].

The results showed convincing evidence for an association between BD and hypertension, suggestive evidence for dementia, Parkinson's disease, and obesity, and weak evidence for asthma, toxoplasmosis, breast cancer, and type 2 diabetes. No associations were confirmed for stroke, myocardial infarction, pain, or irritable bowel syndrome. The authors’ choice of a credibility framework, instead of GRADE, was aligned with the predominantly observational nature of the evidence and the need for a statistically rigorous and reproducible method to account for heterogeneity and publication bias in large‐scale meta‐analyses.

### 3.3 Recommendations for Grading Certainty of Evidence in URs

The recommendations presented in this section were developed through consultation with eight expert methodologists, all of whom are members of the JBI umbrella review methodology group. Consultation was conducted iteratively across three consensus meetings. Experts were purposively selected to represent diverse methodological backgrounds, including evidence synthesis, clinical epidemiology, health services research, and guideline development. Each expert independently reviewed the draft recommendations and provided structured feedback on their clarity, comprehensiveness, and applicability across different UR types. Areas of disagreement were resolved through discussion and majority consensus. Experts unanimously agreed that framework selection should be guided by study type and decision context, and that neither GRADE nor credibility frameworks should be applied universally without consideration of the underlying evidence base.

Based on the findings of the above case studies and our work, GRADE and credibility assessment frameworks are suited to different study designs and review objectives within URs. Framework selection should be tailored to study type and decision context. For interventional URs or where guideline translation is intended, GRADE is preferred. For predominantly observational URs, credibility frameworks can be used to provide reproducible grading, but must be accompanied by context‐aware considerations (risk of bias, plausibility, confounding, and heterogeneity).

Avoid binary interpretations of statistical significance. Report uncertainty transparently, emphasizing prediction intervals, sensitivity analyses, and the potential impact of small‐study effects and excess significance. The steps below detail the methods for using GRADE.

Step 1: Quantify and manage overlapping primary studies.

Before synthesizing certainty ratings across meta‐analyses, calculate the corrected covered area (CCA) for each exposure–outcome pair to quantify the degree of primary study overlap. A CCA of 0%–5% is considered slight, 6%–10% moderate, 11%–15% high, and above 15% very high. Where overlap is moderate or higher, avoid treating each meta‐analysis as an independent source of evidence. Instead, apply the following prioritization rules to select the primary meta‐analysis for each question: (a) prefer the most recently published meta‐analysis, as it is most likely to include the largest sample and most current data; (b) where recency is comparable, prefer the meta‐analysis with the largest number of primary studies or participants; (c) where both are comparable, prefer the meta‐analysis with the more rigorous methodological quality as assessed by AMSTAR 2 or equivalent; and (d) retain additional meta‐analyses as sensitivity sources only, reporting their ratings alongside the primary estimate to demonstrate robustness [5, 21, 22]. All overlap calculations and prioritization decisions should be documented transparently in a supplementary table.

Step 2: Classify and characterize discordant certainty ratings.

When two or more high‐quality meta‐analyses addressing the same question yield conflicting certainty or credibility ratings, the nature of the discordance should first be classified before attempting resolution. Three types of discordance are common and require different responses [23]. First, discordance arising from different included studies (i.e., different search dates or eligibility criteria) is best resolved by identifying the most up‐to‐date or inclusive analysis and treating the others as historical comparators. Second, discordance arising from different analytical methods applied to the same studies (e.g., fixed‐ vs. random‐effects, different heterogeneity estimators) should be addressed through sensitivity analysis, reporting results under both approaches. Third, discordance arising from different framework application (e.g., one meta‐analysis used GRADE and another used a credibility classification) should not be resolved by combining ratings; instead, the two ratings should be presented in parallel with explicit acknowledgement that they measure different constructs and are not directly comparable. In all cases, the source and nature of discordance should be reported explicitly rather than suppressed or averaged [3, 24].

Step 3: Derive an overall umbrella‐level conclusion.

Forming an overall umbrella‐level conclusion requires integrating the certainty or credibility ratings from the prioritized meta‐analyses into a coherent evidence statement. We recommend the following hierarchy of approaches. Where a single prioritized meta‐analysis has been identified (following Step 1), its rating serves as the primary umbrella‐level certainty estimate. Where multiple non‐overlapping or minimally overlapping meta‐analyses are retained, an overall conclusion should be derived by adopting the rating of the highest‐quality meta‐analysis as the anchor, and then considering whether the remaining analyses collectively shift confidence upward (consistent findings across independent samples) or downward (persistent inconsistency or methodological concerns). The overall conclusion should never assign a certainty level higher than that supported by the weakest link in the evidence chain for that question. Where certainty or credibility ratings conflict and cannot be reconciled through the steps above, the appropriate umbrella‐level conclusion is to report the evidence as inconclusive for that exposure–outcome pair, specifying the nature and likely source of conflict. This approach reporting inconclusive evidence explicitly rather than defaulting to the most optimistic rating preserves the integrity of the synthesis and provides more actionable guidance for future research prioritization than a forced consensus would [2, 5, 25].

It is also recommended to maintain separation when both frameworks are used in mixed‐design URs (do not blend outputs) and provide clear rationale for framework choice and document any threshold adaptations for rare outcomes or small fields. We propose the following guidance to support consistent grading of evidence in URs, aligned with prevailing practices for both observational and interventional data as shown in Table 3 [3].

**TABLE 3:** Grading the evidence in URs using either GRADE or credibility assessment frameworks.

Step	Action (credibility framework)	Key results (credibility framework)	GRADE (interventional or mixed URs)
1. Identify eligible meta‐analyses	Select meta‐analyses evaluating exposure–outcome associations	Must report effect sizes (OR, RR, HR), 95% CI, *p* values, number of studies, sample size, heterogeneity (*I* ^2^), bias metrics.	Select meta‐analyses of RCTs or mixed designs; confirm each reports effect estimates, CIs, and sufficient study‐level detail to support domain‐level GRADE judgements.
2. Extract key statistical information	Collect essential data for credibility grading	Extract random‐effects *p* value, number of cases/participants, *I* ^2^, 95% prediction interval, Egger's test (small‐study effects), excess significance test, and largest study significance.	Extract number and design of primary studies, pooled effect estimate and 95% CI, risk of bias assessment (e.g., RoB 2, ROBINS‐I), between‐study heterogeneity (*I* ^2^), assessment of indirectness, and any existing GRADE ratings from included reviews.
3. Apply statistical thresholds for credibility classification	Grade associations using predefined criteria	Class I (Convincing): *p* < 10^−^^6^, >1000 cases, significant largest study, *I* ^2^ < 50%, prediction interval excludes null, no bias evidence. Class II (Highly suggestive): *p* < 10^−^^6^, >1000 cases, but not all bias/consistency criteria met. Class III (Suggestive): *p* < 0.001 and sufficient sample size. Class IV (Weak): Statistically significant but not meeting higher thresholds. Not significant: *p* ≥ 0.05. Thresholds vary across URs (e.g., *p* value cut‐offs, case definitions).	Apply the five GRADE domains to each pooled estimate: (1) risk of bias: downgrade if most primary studies are at high/serious risk; (2) inconsistency: downgrade if *I* ^2^ >50% or prediction interval crosses null; (3) indirectness: downgrade if populations, interventions, or outcomes differ from the decision context; (4) imprecision: downgrade if CI is wide or sample size is insufficient; (5) publication bias—downgrade if small‐study effects or asymmetric funnel plots are evident. Start at high certainty for RCT‐based estimates; start at low for observational. Upgrade observational evidence if large effect, dose–response, or residual confounding unlikely.
4. Conduct sensitivity analyses (optional but recommended)	Test robustness of findings	Alternative models (e.g., Hartung‐Knapp‐Sidik‐Jonkman, DerSimonian‐Laird), subgroup analyses, exclude outliers. Use tools like metaumbrella R package for automation.	Re‐examine GRADE ratings after excluding high‐risk‐of‐bias studies; check whether certainty ratings change when restricting to the most recent or most comprehensive meta‐analysis per question.
5. Report results transparently	Present data and classifications clearly	Tables should include effect sizes, CI, sample size, *I* ^2^, bias indicators, and credibility class. Justify any threshold adaptations.	Present a GRADE SoF table or evidence profile; report the certainty rating (High/Moderate/Low/Very Low) and the key reason for downgrading for each outcome.
6. Interpret findings in context	Go beyond statistics	Consider clinical relevance, biological plausibility, and consistency across studies for evidence‐based application.	Translate certainty ratings into decision‐relevant language; consider magnitude of effect, patient values, and resource implications alongside certainty when forming umbrella‐level conclusions or recommendations.

Abbreviations: CI, confidence interval; GRADE, Grading of Recommendations Assessment, Development and Evaluation; HR, hazard ratio; RCTs, randomized controlled trials, RoB 2, Version 2 of the Cochrane risk‐of‐bias tool for randomized trials; OR, odds ratio; ROBINS‐I, Risk Of Bias In Non‐randomized Studies of Interventions; RR, risk ratio (also called relative risk); SoF, Summary of Findings; URs, umbrella reviews.

## 4 Discussion

As URs grow in prominence for synthesizing observational studies, robust approaches to evaluating evidence certainty have become essential [26]. Traditional tools like GRADE, while accepted for randomized controlled trials, are challenging to apply to URs of non‐interventional data.

Credibility assessment frameworks offer key advantages for URs of observational meta‐analyses. They align closely with statistical outputs typically reported in meta‐analyses—*p* values, heterogeneity, prediction intervals, and bias tests—that are readily available and directly applicable. In contrast, GRADE requires detailed assessment of individual primary studies, data often inaccessible in URs. Credibility frameworks are more objective and reproducible, using predefined thresholds that reduce subjectivity and reviewer variability. Their scalability enables efficient grading across dozens or hundreds of exposure‐outcome associations, while GRADE's rigorous application at this scale is resource‐intensive [6].

GRADE remains valuable for intervention‐focused research where methodological rigor and clinical relevance are essential. Its structured approach across five domains is particularly suited for evidence intended to inform clinical practice or policy.

Both frameworks have limitations. GRADE often downgrades observational evidence regardless of strength or consistency, potentially underestimating high‐quality non‐randomized research. Credibility assessments focus solely on statistical thresholds, neglecting clinical relevance, biological plausibility, and population applicability. The growing adoption of credibility assessment reflects its methodological appropriateness for observational research evaluation. Both methods require expert interpretation and understanding of their assumptions. While GRADE remains the gold standard for interventions, credibility assessment may provide a tailored, efficient method for grading evidence in URs of non‐interventional studies.

This study has several limitations. First, our findings are dependent on the quality and completeness of reporting in the included URs. Second, retrospective application of grading frameworks may introduce subjectivity, particularly where primary data are limited. Third, while our recommendations are broadly applicable, adaptations may be required for specialized fields such as rare diseases or qualitative evidence synthesis. Finally, publication bias and selective reporting in the underlying literature may affect the generalizability of our conclusions.

GRADE and credibility assessment frameworks serve distinct roles in evidence synthesis, with appropriateness determined by study type and intended application. GRADE remains the gold standard for intervention studies and clinical recommendation development, where comprehensive assessment of methodological quality and contextual factors is essential. Credibility assessment frameworks may be more contextually appropriate for URs of observational studies due to their alignment with meta‐analytic outputs, objectivity, and scalability. They utilize readily available statistical measures and predefined thresholds, enabling consistent application across diverse research domains. Rather than competing methodologies, future evidence assessment may benefit from hybrid approaches that combine statistical rigor with contextual considerations, representing a maturation of evidence synthesis methodology tailored to different research questions and applications.

## Conflicts of Interest

All authors are part of the JBI umbrella review methodology group. The authors declare no conflicts of interest.
